# Fragment-based drug discovery: A graphical review

**DOI:** 10.1016/j.crphar.2025.100233

**Published:** 2025-09-10

**Authors:** Dana F. AlKharboush, Frank Kozielski, Geoffrey Wells, Exequiel O.J. Porta

**Affiliations:** a UCL School of Pharmacy, University College London (UCL), London, WC1N 1AX, United Kingdom; b Department of Pharmaceutical Chemistry, Faculty of Pharmacy, King Abdulaziz University, Jeddah, Saudi Arabia

**Keywords:** Drug discovery, Fragment-based drug discovery, FBDD, Fragment-based lead discovery, Fragment screening, Structural biology, Undruggable targets

## Abstract

Three decades after its introduction, fragment-based drug (or lead) discovery (FBDD or FBLD) has become a mature and powerful strategy for generating novel leads, offering distinct advantages for challenging or previously “undruggable” targets where traditional screening (e.g., high throughput screening) often fails. The FBDD approach identifies low molecular weight fragments (MW < 300 Da) that bind weakly to a target; these interactions are detected using highly sensitive biophysical methods such as NMR, X-ray crystallography, and SPR. These initial hits are then optimised into potent leads through structure-guided strategies, including fragment growing, linking, or merging. This graphical review illustrates the modern FBDD workflow, highlighting the critical integration of experimental and computational methods. We discuss how innovations in library design, hybrid screening platforms, and the application of AI/ML are accelerating discovery cycles and improving hit validation. The power of this approach is demonstrated through case studies of FDA-approved drugs, including Vemurafenib and Venetoclax, which progressed from simple fragments to transformative medicines. Finally, we provide an outlook on the future of FBDD as it continues to evolve with emerging technologies to push the boundaries of drug discovery.

## Highlights

• FBDD identifies low-MW fragments by biophysical/structural methods and optimises them to leads.
• FBDD samples chemical space efficiently; >50 fragment-derived compounds entered clinical development.
• Computational tools (docking, FEP, AI/ML) guide fragment growth, complementing screens and speeding optimisation.
• Hybrid platforms combine biophysical and AI/ML to enhance hit discovery and filter artefacts.
• FBDD has delivered approved drugs (e.g., Vemurafenib, Venetoclax) and advanced clinical candidates.

## Graphical abstract

**Figure:**
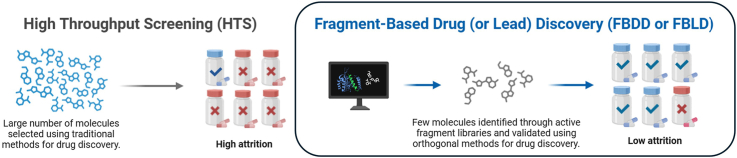

## 1 Introduction

Fragment-based drug discovery (FBDD) has become a cornerstone of modern drug development, facilitating the identification and optimisation of novel leads with remarkable chemical diversity and efficiency (Holvey et al., 2025). While subtle distinctions exist, in this graphical review, the term will be used interchangeably with fragment-based lead discovery (FBLD). In FBDD, very small molecules (“fragments”), typically adhering to the “Rule of 3” (MW ≤ 300, logP ≤ 3, ≤3 hydrogen bond donors/acceptors), are screened for weak binding, with promising hits subsequently grown, linked, or merged to generate potent leads (Li, 2020; Shuker et al., 1996). The simplicity of fragments confers high ligand efficiency and allows greater scope for optimisation compared to traditional high-throughput screening (HTS) hits. The value of FBDD was demonstrated in the late 1990s by the “SAR by NMR” approach (Shuker et al., 1996), and, by the 2000s, pioneering examples had established FBDD as a powerful, complementary alternative to HTS (Chessari and Woodhead, 2009; Hajduk and Greer, 2007). This success was largely due to its significantly higher hit rates, which are often in the order of 5 %–20 %, compared to less than 1 % for HTS (Foley et al., 2021). Early reviews documented its rapid ascent, as fragment-derived leads entered trials and advanced into the clinic (Chessari and Woodhead, 2009).

Fragments excel at efficiently sampling chemical space: a library of only 500–3000 compounds can cover broad chemotype diversity (Khedkar et al., 2025). Their small size enables them to occupy cryptic pockets and other challenging sites, such as protein-protein interfaces and allosteric sites (Bon et al., 2022; Xu and Kang, 2025; Khedkar et al., 2025). While shallow binding pockets remain a significant challenge for any small molecule, covalent ligands derived from fragments have the potential to achieve improved potency for these sites (Lu et al., 2021; McCarthy et al., 2024). This covalent fragment approach has proven successful in targeting previously intractable pockets, as exemplified by the discovery of inhibitors for KRAS G12C (*vide infra*) (Mathieu et al., 2022). Although their binding affinities are typically weak (high μM–mM K_D_), these hits are highly ligand-efficient and serve as tractable starting points. Importantly, fragment hits frequently represent novel chemotypes; careful optimisation can yield drug-like leads with improved selectivity. The success of FBDD is reflected in the expanding pipeline: by 2016, over 30 fragment-originated clinical candidates and two drugs had emerged (Erlanson et al., 2016). Today, the number exceeds 50 clinical candidates and at least seven approved drugs, covering oncology and infectious diseases (Khedkar et al., 2025).

**Fig. 1:**
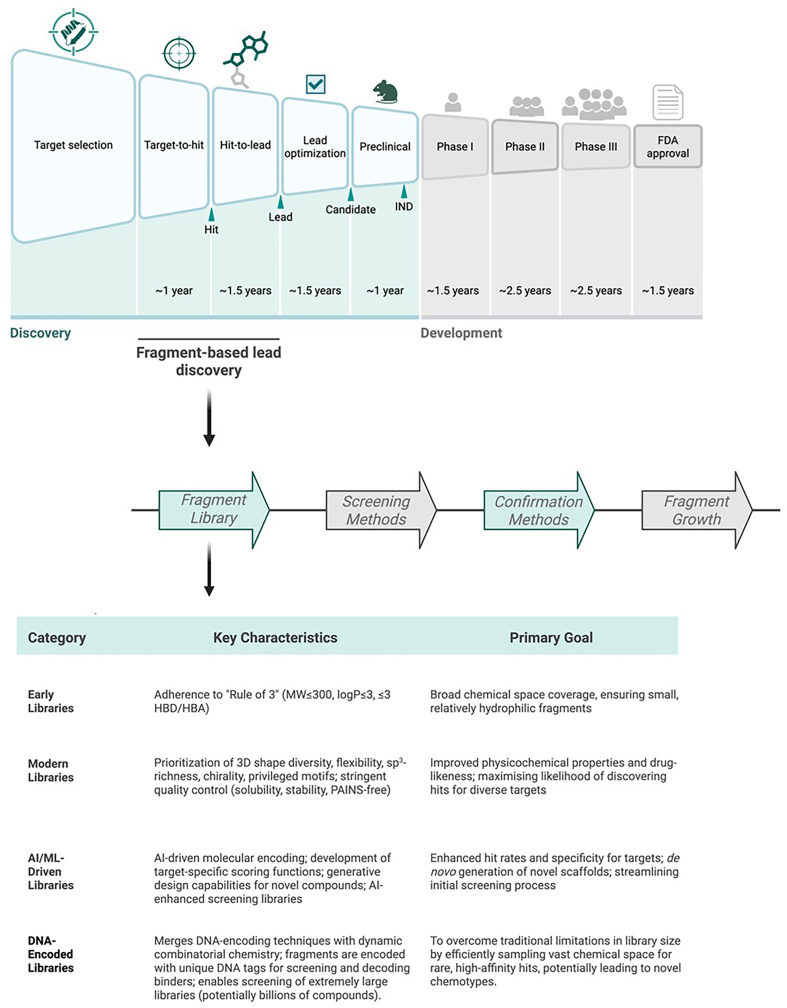
An overview of the drug discovery and development pipeline, highlighting the integration of the FBDD workflow at the early stages. The FBDD process begins with a fragment library and proceeds through screening, confirmation, and fragment growth to generate hit compounds. The table categorises different types of fragment libraries based on their key characteristics and primary goals, including early libraries adhering to the “Rule of 3”, modern libraries prioritising 3D shape diversity, and advanced libraries driven by AI/ML or encoded with DNA.

Detecting very weak binders reliably remains the main technical challenge. Fragment screening requires highly sensitive methods with low false-positive rates (Holvey et al., 2025). Fragment hits, once identified, must be efficiently optimised from low starting affinities, relying on robust medicinal chemistry and structural insights. Stringently curated libraries, composed of soluble, non-reactive, and pan-assay interference compound (PAINS)-free fragments, are critical (Bon et al., 2022). The continued evolution of library design, screening technologies, and optimisation strategies has driven ongoing improvements. Below, we detail the essential steps of FBDD: from library design and screening to hit validation, optimisation, and emerging advances (Fig. 1).

## 2 Fragment library design

A well-designed fragment library is foundational for FBDD success (Fig. 1). Early libraries followed the “Rule of 3” (*vide supra*), ensuring small, relatively hydrophilic fragments (Congreve et al., 2003). Modern libraries prioritise 3D shape diversity and flexibility (de Esch et al., 2022), a trend known as “escaping flatland”, with the goal of improving physicochemical properties, reducing promiscuity, and accessing novel binding modes. However, a recent analysis suggests this trend should be balanced, as the link between higher 3D character (Fsp^3^) and clinical success has not persisted for drugs approved after 2009 (Churcher et al., 2025). This “return to flatland” highlights that flatter, sp^2^-rich fragments remain highly valuable and successful starting points, particularly as 3D-enriched libraries may yield lower hit rates for challenging targets. Therefore, diversity is key: effective libraries sample a wide range of chemotypes, including both sp^2^-and sp^3^-rich scaffold frameworks, to maximise the likelihood of discovering hits for different targets. Over time, inclusion of chiral and privileged fragments (motifs recognised to bind specific pockets) has grown (Khedkar et al., 2025).

Fragments can be tailored for particular target classes; for instance, covalent cysteine-binding fragments for kinases or G protein-coupled receptors (GPCRs) (Lu et al., 2021). Recently, covalent fragment-based discovery has become an increasingly popular and successful strategy (Keeley et al., 2020), driven in part by the clinical success of targeted covalent inhibitors (Singh, 2022). This approach typically involves either screening libraries of fragments already equipped with a reactive “warhead” (usually an electrophilic group) or appending a reactive moiety to a noncovalent fragment hit during optimisation (Forrest and Parker, 2023; Holvey et al., 2025). While cysteine is the most frequently targeted amino acid, recent efforts have expanded the toolkit to include warheads that can react with other residues, such as lysines, tyrosines, and serines, broadening the range of proteins that can be targeted (Lu et al., 2021; Csorba et al., 2023). These strategies have proven highly effective, even in cell-based screens, for discovering potent and selective inhibitors for challenging targets, including enzymes and protein-protein interactions (Erlanson et al., 2019; Holvey et al., 2025; Niphakis and Cravatt, 2024; Porta et al., 2025). A key advantage of the covalent approach is that it greatly facilitates computational modelling; knowing the attachment point on the protein provides a strong anchor for predicting the binding pose of the fragment and guiding subsequent optimisation (Lu et al., 2021).

Fragments must be “screenable”: highly soluble (to enable high-concentration assays) (O'Reilly et al., 2019), chemically stable and tractable (to allow rapid follow-up progression), and free from aggregation or non-specific binding. Rigorous quality control removes problematic compounds, such as PAINS. Keserű et al. (2016) surveyed pharmaceutical fragment libraries and emphasised the need for continual refinement as new design principles emerge. Excluding overly lipophilic fragments is important, since growing fragments typically increases hydrophobicity; starting with polar fragments supports retention of drug-like properties. Metrics such as ligand efficiency (LE, which focuses on the potency of a compound relative to its molecular size) and ligand efficiency-dependent lipophilicity (LEAT, which builds upon LE by incorporating lipophilicity into the efficiency calculation) guide prioritisation of hits with optimal potency and physicochemical balance (Erlanson et al., 2016). Contemporary libraries often blend commercial and custom fragments. Interest in dynamic or tethered fragments (e.g., reversibly covalent fragments, fragment merging pools) is rising to further expand accessible chemical space. Ultimately, a high-quality fragment library is compact but chemically diverse, populated by “clean” compounds to maximise novel binder discovery.

Beyond traditional fragment collections, DNA-encoded libraries (DELs) represent a powerful and highly diverse source of chemical matter for screening (Gironda-Martínez et al., 2021). A DEL is a high-throughput screening technology that employs unique DNA tags to encode and identify vast collections of small molecules, enabling the rapid discovery of binders to biological targets. Briefly, DELs link each small molecule to a distinct DNA barcode, allowing for efficient selection, amplification, and sequencing of hits from screens involving millions to billions of compounds (Zhao et al., 2024). However, analysing DEL data can be challenging, particularly for identifying weak binders from the high hit rates often generated (Foley et al., 2021).

Finally, virtual screening of fragment libraries is now routinely performed alongside experimental campaigns, typically using molecular docking and molecular dynamics simulations to identify and prioritise hits, respectively (Togre et al., 2022). However, the reliability of these predictions can be hampered by inaccuracies in the underlying force fields and scoring functions used to approximate complex binding interactions (Hong et al., 2024). To overcome these challenges, machine learning (ML) models are increasingly being integrated to enhance fragment scoring and predict promising analogues. While these advanced computational methods are dramatically improving the efficiency of screening (Jinsong et al., 2024; Lehasa and Chude-Okonkwo, 2024; Yoo et al., 2025), their prospective application is still developing, with many current models yet to transition from benchmarking exercises to consistent success in real-world drug discovery campaigns.

## 3 Fragment screening strategies

**Fig. 2:**
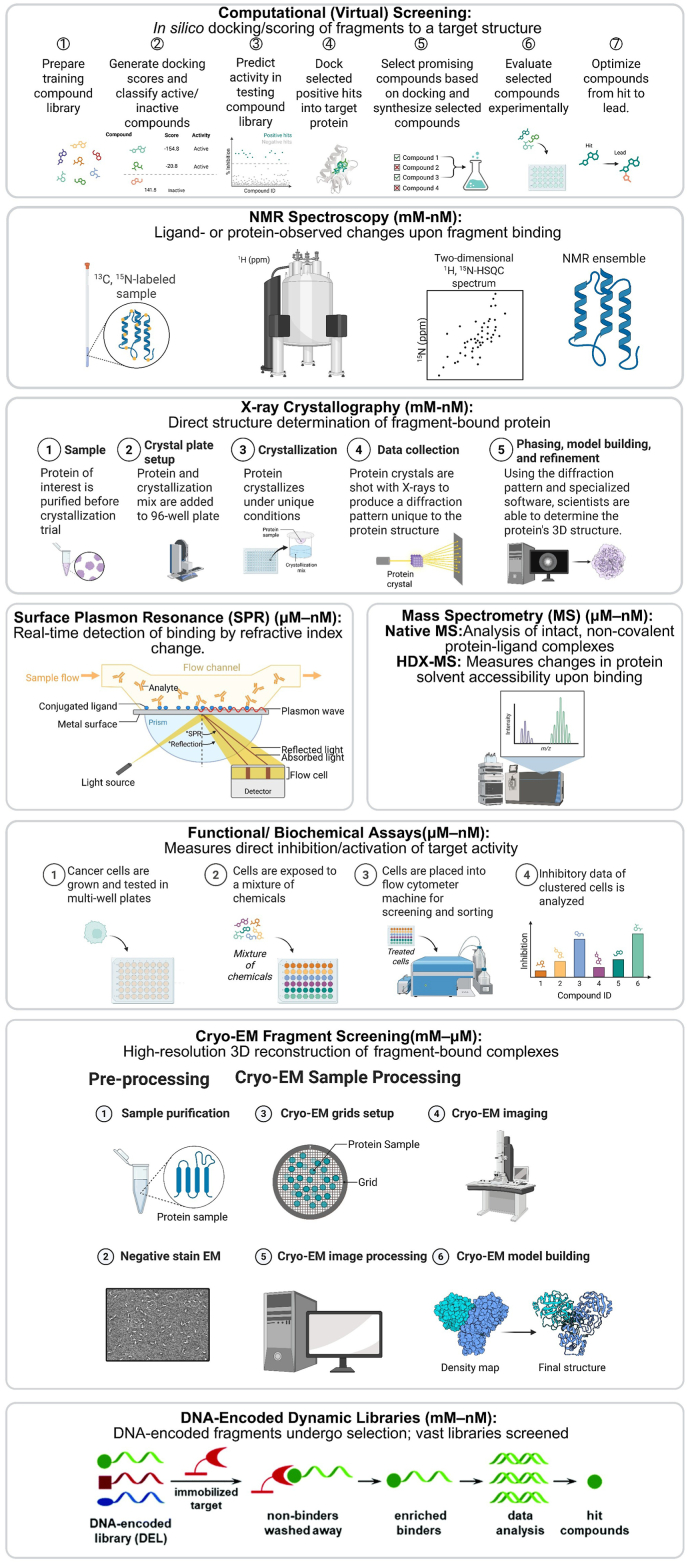
A summary of the principal experimental and computational screening and confirmation methods employed in FBDD. The figure illustrates sensitive biophysical and structural techniques, including NMR Spectroscopy, X-ray Crystallography, SPR, MS, and Cryo-EM. They are used to detect and characterise weak fragment binding. These are complemented by computational (virtual) screening, functional assays, and DEDLs. An integrated, orthogonal approach enhances the reliability of hit discovery and validation, guiding the structure-based optimisation of fragments into potent, drug-like leads.

Because fragment binding affinities are typically weak, specialised biophysical screening methods are necessary (Fig. 2, Table 1).

**Table 1:** Comparison of Key Screening and Confirmation Methods in FBDD. A summary of the principal experimental and computational methods employed in FBDD. The table outlines a range of sensitive biophysical and structural techniques used to detect and characterise weak fragment binding. These are complemented by functional assays and advanced computational approaches such as virtual screening and machine learning (ML), each with distinct advantages and limitations for hit discovery and validation.

Screening Strategy	Use	Advantages	Limitations	Key Considerations
NMR Spectroscopy	Identifies weak fragment hits; maps binding sites; elucidates ligand orientation	Highly sensitive; atomic-level binding info; suitable for mix-and-screen (e.g.,^19^F NMR)	Requires isotopic labelling; specialised expertise and hardware; limited binding pose detail	Ideal for initial hit identification; fragment affinity ranking; epitope mapping
X-ray Crystallography	Direct and unambiguous identification of binders and binding poses; reveals cryptic sites	High-resolution structural data; directly guiding medicinal chemistry	“Limited throughput”; requires crystallisable protein	“Gold standard” for hit validation and structure-guided optimisation
SPR	Ranks fragments; determines kinetic and affinity parameters	Label-free; real-time kinetics and affinity data; high throughput; suitable for mixtures	Requires immobilisation of binding partner; sensitive to non-specific binding	Widely used for affinity ranking and kinetic characterisation (K_D_, k_on_, k_off_); Hit validation
ITC	Directly measures binding thermodynamics (enthalpy and entropy)	Label-free, in-solution measurement; provides a complete thermodynamic profile of binding	Low throughput; requires large amounts of pure protein; not suitable for very weak or very tight binders	Widely used for validating moderate-to-strong interactions and understanding the thermodynamic drivers of binding
Native MS	Intact analysis of protein–ligand complexes in gas phase; characterises binding	High sensitivity, adaptable for high-throughput; Label-free; stoichiometry and affinity data; minimal sample requirement	Artifact-prone without careful handling; lacks direct pose information; challenging with heterogeneous samples; optimisation required to preserve (noncovalent) complexes	Complements structural methods; confirms binding and stoichiometry
HDX-MS	Analyses protein conformational dynamics and solvent accessibility upon binding	Reveals conformational changes; indirect binding site/allosteric insights	Indirect binding mapping; moderate throughput; requires careful design and interpretation; complex data analysis	Valuable for allosteric mechanisms and mechanistic understanding
Functional/Biochemical Assays	Screens for target inhibition or activation	Direct biological relevance (e.g., measure of biological activity); links activity to therapeutic effects; ABPP technology	Fragments often too weak for detection	Early indication of functional effect, Validating optimised leads or highly sensitive readouts
Computational (Virtual) Screening	Predicts binders via *in silico* docking/scoring	High throughput; cost-effective; no protein required; prioritises fragments (and experimental screening)	Dependent on accurate scoring functions and protein models; potential false positives	Essential in guiding fragment prioritisation and growth; scaffold hopping, merging, linking design
ML/AI-Driven Screening	ML/AI-based prioritisation and de novo fragment design	Rapid, scalable analogue prioritisation; novel scaffold generation; improved hit rates	Requires large datasets; some methods lack interpretability (“black box”); emerging technology	Transformative for efficiency, design, and library optimisation
Cryo-EM Fragment Screening	High-resolution structures of challenging proteins (large, flexible, membrane-bound)	Suitable for difficult-to-crystallise targets; high-resolution structural insights	Specialised hardware/expertise; computationally intensive; emerging technology (still evolving)	Expands target space significantly (e.g., GPCRs, ion channels)
DNA-Encoded Dynamic Libraries (DEDLs)	DNA-encoded fragments undergo selection; vast libraries screened	Screens billions of compounds; detects rare, high-affinity hits	Complex library synthesis; potential for DNA artifacts	Ultra-high throughput screening; novel chemotype discovery

### 3.1 NMR spectroscopy

NMR Spectroscopy is a highly sensitive method for detecting weak (mM K_D_) fragment binding (Kirsch et al., 2019). Ligand-observed techniques (e.g., by STD-NMR, WaterLOGSY, ^19^F) are particularly advantageous as they do not require modification of the protein and can detect low-occupancy binding events at lower ligand concentrations, reducing concentration-related artifacts. For example, ^19^F NMR provides distinct signals with little background, allowing mix-and-screen workflows (Buchholz and Pomerantz, 2021; Li, 2020; Wu et al., 2025). Protein-observed methods (e.g., HSQC) offer more direct, atomic-level information on binding sites and ligand orientation but typically require expensive, isotopically labelled protein (e.g., with ^15^N and/or ^13^C) and specialised hardware facilities. NMR spectroscopy, while central to the original “SAR by NMR” approach (Erlanson et al., 2016), often lacks the detailed 3D structural information provided by techniques like X-ray crystallography (Li, 2020).

### 3.2 X-ray crystallography

X-ray crystallography is a gold standard for FBDD (Collins et al., 2018). Soaking or co-crystallising crystals of the target protein with fragments, followed by determining their structures, allows direct identification of binders and their poses (Holvey et al., 2025). X-ray fragment screening reveals binding hotspots and cryptic sites. For instance, fragment-bound Hepatitis C virus non-structural protein 3 protease/helicase structures revealed novel inter-domain pockets (Saalau-Bethell et al., 2012). Though throughput is limited to a few 100 datasets per day, X-ray screening often gives unambiguous results and guides chemistry directly. To address this limitation, high-throughput crystallographic fragment screening has been pioneered at specialised synchrotron facilities (e.g., XChem in the UK) (Fearon et al., 2025; Krojer et al., 2020). These platforms have made the technique more accessible and capable of screening thousands of fragments rapidly, providing rich structural data for numerous projects. A key innovation enabling this is the integration of advanced computational tools like the PanDDA algorithm, which reliably detects weak, low-occupancy fragment binding that is often missed by conventional analysis (Pearce et al., 2017). This combination of high-throughput data collection and sensitive analysis was critical to the success of major initiatives, including the rapid development of inhibitors for the SARS-CoV-2 main protease in the “COVID Moonshot” project (Boby et al., 2023).

### 3.3 Cryo-electron microscopy (cryo-EM)

Cryo-EM is an emerging technique for fragment screening that has not yet reached maturity for routine application. Cryo-EM can determine the structures of protein-fragment complexes, which is particularly useful for large, dynamic, or membrane-bound targets that resist crystallisation (Van Drie and Tong, 2020). While initially demonstrated as a proof-of-concept, more recent work has validated its use for routine fragment screening by enabling high-throughput structure determination for challenging targets like membrane proteins (Saur et al., 2020; Reeks et al., 2025). However, significant challenges remain. Unlike X-ray crystallography where the crystal lattice restricts ligand conformations, the dynamic nature of particles in cryo-EM presents considerable difficulties for fragment screening. The process requires high fragment occupancy, and a consistent binding pose across particles to avoid averaging out the signal during image processing and 3D reconstruction (Renaud et al., 2018). These stringent requirements, combined with the complexities of image triage and clustering, mean that cryo-EM is not yet a routine or gold-standard method for primary fragment screening. Besides, a related technique, Microcrystal Electron Diffraction (MicroED), has also been successfully used to identify fragment hits by applying electron diffraction to nanocrystals, thereby expanding the toolkit for structurally characterising weak binders (Clabbers et al., 2020). For cryo-EM to become more widely adopted in FBDD, a promising future direction is the integration of computational methods with lower-resolution maps (3.5–4.5 Å) (Kaur et al., 2021), where molecular docking and ML can help accurately place fragments when the experimental density is ambiguous to guide drug design.

### 3.4 Mass spectrometry (MS)

MS-based screening encompasses several techniques. Denaturing methods like affinity selection–MS (AS-MS) and hydrogen–deuterium exchange MS (HDX–MS) are used to identify hits or map binding sites, respectively (Sternicki and Poulsen, 2024). A particularly powerful technique for FBDD is native MS (nMS), which directly detects intact, noncovalent fragment–protein complexes. This allows for the rapid determination of binding stoichiometry, which helps differentiate specific from non-specific binding and reduces false positives. Furthermore, nMS can be used to calculate binding affinities (K_D_) and may preferentially detect enthalpically-driven interactions, which are often considered higher-quality starting points for optimisation (Sternicki and Poulsen, 2024). While all MS methods offer high sensitivity with minimal protein consumption, nMS provides the distinct advantage of directly observing the specific binding event in a near-native state. More recently, MS-proteomic methods have also been successfully applied to covalent fragment screening across entire proteomes, allowing for broad profiling of fragment reactivity and selectivity (Forrest and Parker, 2023).

### 3.5 Functional/biochemical assays

Functional/Biochemical assays are a foundational screening method in FBDD campaigns, consistently representing a significant portion of hit-finding strategies over the past decade (Holvey et al., 2025). While the weak affinity of most fragments presents a challenge, these assays can be successfully applied when they are sensitive enough to detect hits, often at high ligand concentrations. A powerful example of this is Activity-Based Protein Profiling (ABPP), a functional chemoproteomic technique used to monitor the activity of entire enzyme families (Porta and Steel, 2023), which has been successfully adapted for covalent FBDD (Niphakis and Cravatt, 2024). Moreover, this technology (i.e., ABPP) serves as the basis for high-throughput screening platforms capable of rapidly evaluating fragment and small-molecule libraries with high sensitivity (Porta et al., 2025). Although other biophysical methods are also prevalent, the continued use of biochemical assays highlights their enduring importance in identifying initial fragment hits.

### 3.6 Computational (virtual) screening

Docking fragments *in silico* against target proteins helps identify potential binders efficiently (Khedkar et al., 2025; Holvey et al., 2025). Currently, computational (virtual) screening has evolved from screening millions of in-stock compounds to navigating vast “make-on-demand” chemical spaces of over 40 billion molecules (Carlsson and Luttens, 2024). A highly efficient strategy for this is virtual fragment screening, where docking of large fragment libraries (e.g., 14 million compounds) identifies initial hits with impressive accuracy and higher hit rates than comparable experimental screens (e.g., HTS) (Luttens et al., 2025). To manage these immense libraries, ML is now being integrated into the pipeline. ML models trained on initial docking results can prioritise regions of chemical space, reducing the number of compounds needing explicit docking by up to 100-fold (Luttens et al., 2025). This synergy between large-scale docking and ML has proven successful for discovering potent inhibitors for challenging targets, including viral enzymes and GPCRs, by enabling the rapid, structure-guided elaboration of initial fragment hits into submicromolar leads (Luttens et al., 2022).

### 3.7 Other techniques: surface plasmon resonance (SPR), differential scanning fluorescence (DSF), and isothermal titration calorimetry (ITC)

SPR is a widely used, high-throughput technique for primary fragment screening. It is a label-free method that detects real-time binding by measuring refractive index changes on a sensor chip where the target protein is immobilised (Li, 2020; Holvey et al., 2025; Erlanson et al., 2019). This allows for the rapid ranking of fragments and provides valuable kinetic data (k_on_/k_off_), though it can be susceptible to artifacts from non-specific binding (Davis and Erlanson, 2013). Two other common methods, typically used for hit validation rather than primary screening, are DSF and ITC. DSF (also known as Thermal Shift Assay, TSA) is a rapid, high-throughput method that measures changes in protein stability upon ligand binding. However, it is known to have high rates of both false positives and false negatives, making it a less reliable primary assay (Erlanson et al., 2019; Sternicki and Poulsen, 2024). ITC provides a complete thermodynamic profile of binding (ΔG, ΔH, and ΔS), but its low throughput and very high protein consumption make it best suited for validating and characterising moderate-to-strong interactions later in the discovery process (Davis and Erlanson, 2013).

### 3.8 Orthogonal approaches

Using an orthogonal approach (combining multiple complementary screens) is a standard strategy to cross-validates hits, increases confidence, and reduces false positives (Kirkman et al., 2024). For example, a fragment identified by NMR and confirmed by X-ray or SPR is much more likely to be a true binder. Orthogonal screening also helps classify fragments by binding site (e.g., through competition SPR or NMR epitope mapping). A closer examination of the field reveals a clear, synergistic trend: advanced biophysical methods (Cryo-EM for membrane proteins and large complexes, Native MS for intact protein-ligand complexes, HDX-MS for conformational dynamics) are expanding the range of targets that can be physically screened. This synergy is further enhanced by emerging artificial intelligence (AI) models like Boltz2, which can predict binding affinities with an accuracy approaching that of computationally expensive free-energy perturbation (FEP) methods, allowing for rapid *in silico* prioritisation of fragments for experimental validation (Passaro et al., 2025).

### 3.9 DNA-encoded dynamic libraries (DEDLs)

The recent advent of DEDLs has significantly advanced FBDD by DEL (*vide supra*) with dynamic combinatorial chemistry, overcoming traditional limitations in library size, and facilitating the detection of rare but high-affinity fragment binders (Zhao et al., 2024).

### 3.10 Synthetic tractability and other considerations

Considering synthetic tractability is crucial during hit follow-up (St Denis et al., 2021): some fragment hits, especially from high-throughput X-ray screens, are deprioritised due to lack of accessible analogues. Planning synthetic routes early can influence which hits to advance. An innovative approach to this challenge is to evaluate crude reaction mixtures directly using high-throughput crystallography, a method termed Binding-site Purification of Actives (B-SPA) (Grosjean et al., 2025). In this strategy, the protein's binding site effectively “purifies” the active compound from the crude mixture, allowing for the rapid structural evaluation of many parallel multi-step reactions and providing an immediate structure-activity relationship map without the need for traditional purification.

In summary, sensitive biophysical techniques are critical for reliably detecting weakly binding fragments (Coyle and Walser, 2020). The choice of method depends on target properties and resources, and a combination is often used to avoid missing viable fragments. Once hits are identified, they move to the validation and characterisation stage.

## 4 Hit validation and characterisation

Fragment hits require thorough validation, including confirmation by orthogonal methods, estimation of their affinity, and detailed structural information on the target-fragment interaction (Fig. 2).

A typical validation workflow includes:

I. Re-screening hits individually using the primary detection method.
II. Testing in one or more secondary assays (e.g., hits from NMR validated by SPR or crystallography, and vice versa). Consistency across methods is vital, since many initial hits are false positives (Holvey et al., 2025).
III. Crystallography, where possible, is the gold standard. An X-ray co-crystal structure mostly provides unambiguous insight into binding mode and site. Even hits discovered by other methods are often soaked into crystals for validation.
IV. If a crystal structure cannot be obtained, binding can be inferred indirectly through mutational analysis or structure–activity relationship (SAR) by catalogue. This approach involves testing a small set of (in-house and/or commercial) analogues to quickly probe the chemical space around a hit. Advanced strategies now enable this even without purifying the final compounds; for example, through off-rate screening (ORS), where crude reaction mixtures are screened directly using SPR to rank binders based on their dissociation rates (Erlanson et al., 2019).

Typical fragment hits are weak (K_D_ 200 μM–10 mM), but some achieve low μM affinity or ligand efficiency >0.3, marking them as high-quality hits (Khedkar et al., 2025). Additional techniques, like ITC (measuring binding thermodynamics) and thermal shift (ΔT_m_) assays (demonstrating protein stabilisation), complement validation. Another aspect of validation is checking for artifact behaviour: fragments that aggregate, are sticky, or reactive can be weeded out by various experiments. For instance, NMR is a straightforward method to assess compound solubility and aggregation (Ayotte et al., 2019), while detergent-based assays or LC-MS analysis can check for non-specific binding and covalent adducts, respectively (Sternicki and Poulsen, 2024).

Careful library curation is essential because fragment screens are highly susceptible to artifacts. Unexpected behaviours can arise, particularly when fragments are at high concentrations, leading to issues such as aggregation, compound degradation, or interference from low-level impurities (Davis and Erlanson, 2013). Even with rigorous quality control, unexpected behaviours can still arise, making orthogonal hit validation critical. Validated fragments are then clustered by chemotype, and initial SAR are explored. Even a minimal SAR (i.e., testing a few analogues) can confirm pocket specificity and inform optimisation. As fragment screening scales dramatically with high-throughput methodologies such as automated MS, high-throughput X-ray crystallography, and DELs, the concept of validation is evolving from purely experimental, sequential approaches toward integrated workflows that combine computational pre-validation with rapid, orthogonal biophysical checks.

As aforementioned, synthetic tractability is crucial: fragments that can be readily elaborated are prioritised. Polar, unfunctionalised fragments can bind well but may be difficult to optimise (St Denis et al., 2021). Collaboration with medicinal chemists at this stage will help to identify “growth vectors”, i.e., sites for further chemical expansion. Additionally, AI- and LM-assisted computational tools (docking, pharmacophore mapping, molecular dynamics) aid in mapping fragment interactions and suggest strategies for fragment growing, merging, or linking. Teams typically select 2–6 validated hits for further optimisation, based on novelty, tractability, and strategic fit.

## 5 Fragment evolution: growing, merging, linking (and hybrid) approaches

**Fig. 3:**
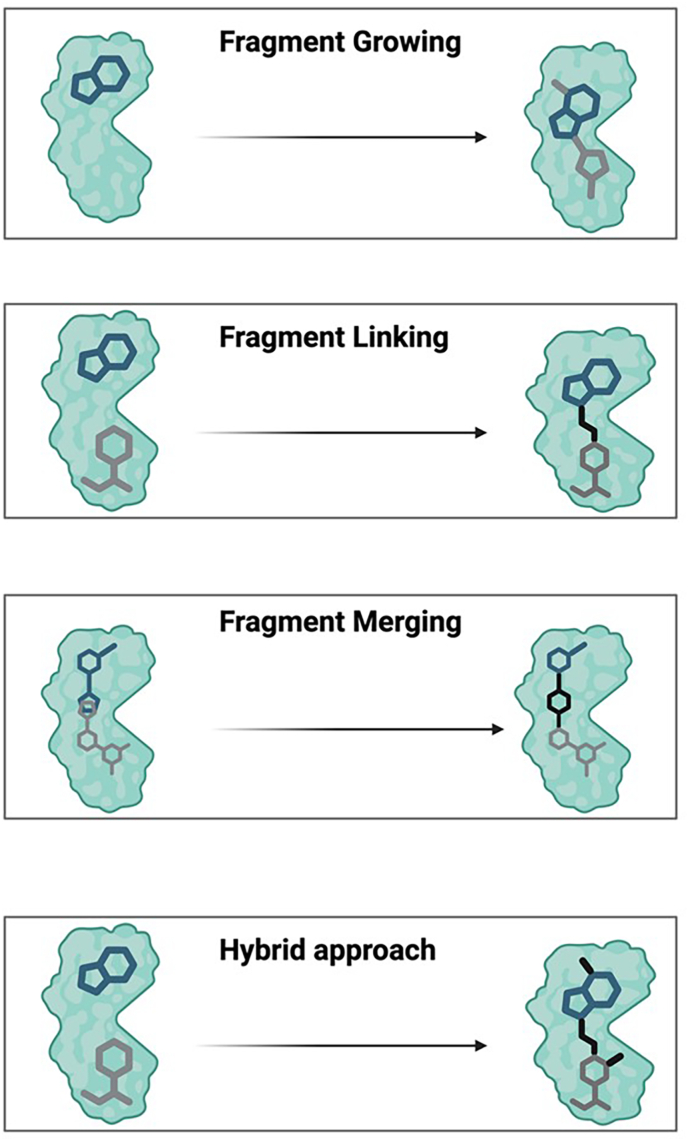
Key strategies for fragment-to-lead (F2L) optimisation. This figure illustrates the primary structure-guided strategies used to evolve low-affinity fragment hits into potent lead compounds. Fragment Growing involves the iterative addition of chemical groups to a fragment to explore adjacent binding pockets and increase affinity. Fragment Linking connects two distinct fragments that bind to proximal sites on the target, creating a single, high-potency molecule. Fragment Merging combines the structural features of two or more overlapping fragments into a novel chemical scaffold that retains the key binding interactions of its predecessors. A hybrid approach (e.g., Linking + Growing) integrates elements from multiple strategies to efficiently achieve the desired potency and drug-like properties.

The evolution of a low-affinity fragment hit into a potent lead compound is a critical phase in FBDD known as fragment-to-lead (F2L) optimisation. This process is typically driven by structure-guided design and employs several key synthetic strategies, including fragment growing, linking, merging, and hybrid approaches (Fig. 3).

### 5.1 Fragment growing

Incremental addition of atoms or groups to the fragment, guided by structural data, to improve complementarity and affinity while retaining the core binding mode. For example, Kirsch et al. (2019) described expanding an imidazole fragment to a triazole inhibitor (IC_50_ 17 μM) by click chemistry, with improved ligand efficiency. Growing can be performed with or without structural information, though structure-guided approaches are more efficient.

### 5.2 Fragment linking

If two fragments bind to adjacent or proximal sites, they can be linked to form a high-affinity molecule. This method, exemplified by BCL-XL inhibitors (*vide infra*), can dramatically boost potency (often 100–1000-fold). However, it is now generally considered the most challenging F2L approach. The difficulty lies in designing a linker that maintains the optimal binding geometry of both original fragments, a task that requires precise structural information and often leads to molecules with complex synthetic routes and less favourable physicochemical properties (Tao et al., 2014; Mathieu et al., 2022). A recent successful example of this challenging strategy was the development of a molecular glue that stabilises the 14-3-3/ERα protein-protein interaction (Visser et al., 2023). In this work, a covalent “tethered” fragment and a noncovalent fragment were co-crystallised, providing precise structural information that guided the design of a linker to create a potent, noncovalent lead while largely conserving the original binding modes of both fragments.

### 5.3 Fragment merging/scaffold hopping

In fragment merging, fragment hits that bind to overlapping sites (or share substructures) are combined (merged) into a single, new chemotype that retains the key interactions of the original fragments (Kirsch et al., 2019). Similarly, scaffold hopping involves replacing the core scaffold while preserving crucial interaction points, a common tactic to improve binding, enhance physicochemical properties, or secure new intellectual property.

### 5.4 Hybrid approaches

Often, multiple strategies are combined, e.g., growing one end of a fragment, linking to another, or merging features. Computational approaches can propose such hybrids (Khedkar et al., 2025). At every step, structural validation is essential to confirm the desired binding mode and ensure efficient progression (Erlanson et al., 2016).

### 5.5 Other considerations

Regardless of the chosen evolution strategy, the entire F2L process is governed by several practical considerations. Throughout the optimisation, core medicinal chemistry principles, such as maintaining favourable metabolic profiles and avoiding toxic motifs, are paramount. Efficiency is closely monitored using metrics like LE and LEAT (*vide supra*) (Erlanson et al., 2016), as declines in LE can signal diminishing returns when increasing molecular size. Furthermore, synthetic tractability remains a critical constraint; sometimes slightly less potent, but more chemistry-friendly fragments are better for progression (St Denis et al., 2021). These principles often favour fragment growing and merging, as fragment linking can present significant challenges in maintaining optimal binding geometry and achieving straightforward syntheses. Finally, a variety of *in silico* methods are crucial for successfully developing lead compounds, including hot spot analysis to identify key interaction areas, SAR by catalogue to explore commercially available analogues, and ML models to filter for desirable ADMET properties (de Souza Neto et al., 2020).

## 6 Selected case studies in FBDD

Three representative cases illustrate the power and importance of FBDD, underscoring the critical role of structural data, meticulous chemistry (such as optimising linkers and functional groups), and a readiness to embrace unconventional strategies like covalent tethering.

### 6.1 B-RAF inhibitor vemurafenib (PLX4032)

**Fig. 4:**
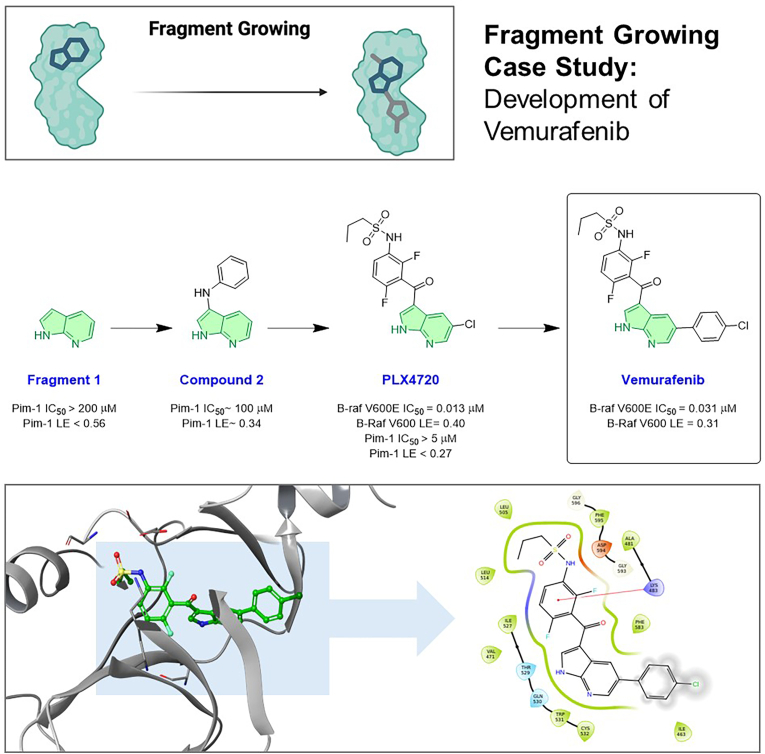
Fragment growing case study: The development of Vemurafenib. This workflow showcases the evolution of a B-RAF kinase inhibitor. The process started with a low-affinity 7-azaindole fragment (Fragment **1**) identified via screening. Through iterative, structure-guided growing, this hit was elaborated by adding functional groups to engage with neighbouring sub-pockets, progressing through intermediates like Compound **2** and PLX4720. This optimisation campaign, guided by X-ray crystallography (PDB ID 3OG7), ultimately yielded the potent inhibitor Vemurafenib, an FDA-approved drug for BRAF-mutant melanoma. The bottom panels illustrate Vemurafenib's binding mode, highlighting the key interactions achieved.

Developed via fragment growing from initial low-affinity hits to a potent drug (Fig. 4). The oncogenic kinase B-RAF-V600E (a driver in melanoma) was one of the first targets tackled by FBDD to yield an FDA-approved drug (Kim et al., 2014). Researchers at Plexxikon performed a fragment screen (using X-ray and biochemical assays) and identified a 7-azaindole fragment (Fragment **1**) that bound in the BRAF active site (Tsai et al., 2008; Bollag et al., 2012). Though weak, this fragment provided a foothold in the ATP-binding pocket. Using iterative structure-guided growing, the team added functional groups to interact with neighbouring sub-pockets. Notably, the fragment and an early analogue were co-crystallised with BRAF to guide design (PDB ID 3OG7). Over multiple cycles, the fragment was elaborated into PLX4032 (Vemurafenib), a ∼500 Da MW inhibitor with nM affinity. Vemurafenib showed unprecedented efficacy in BRAF-mutant melanoma and was approved by the FDA in 2011 (Kim et al., 2014). This case exemplifies fragment growing guided by x-ray crystallography: the initial fragment's binding mode was conserved through the evolution, and by carefully adding rings and substituents, while monitoring kinase selectivity and pharmacokinetics, an oral drug was developed. Vemurafenib's discovery validated FBDD's promise; it was one of the first drugs directly stemming from a fragment hit. Interestingly, a similar azaindole fragment was independently used by AstraZeneca in an AKT kinase project, highlighting that the same fragment can seed multiple leads (Addie et al., 2013).

### 6.2 BCL-2 inhibitor venetoclax (ABT-199)

**Fig. 5:**
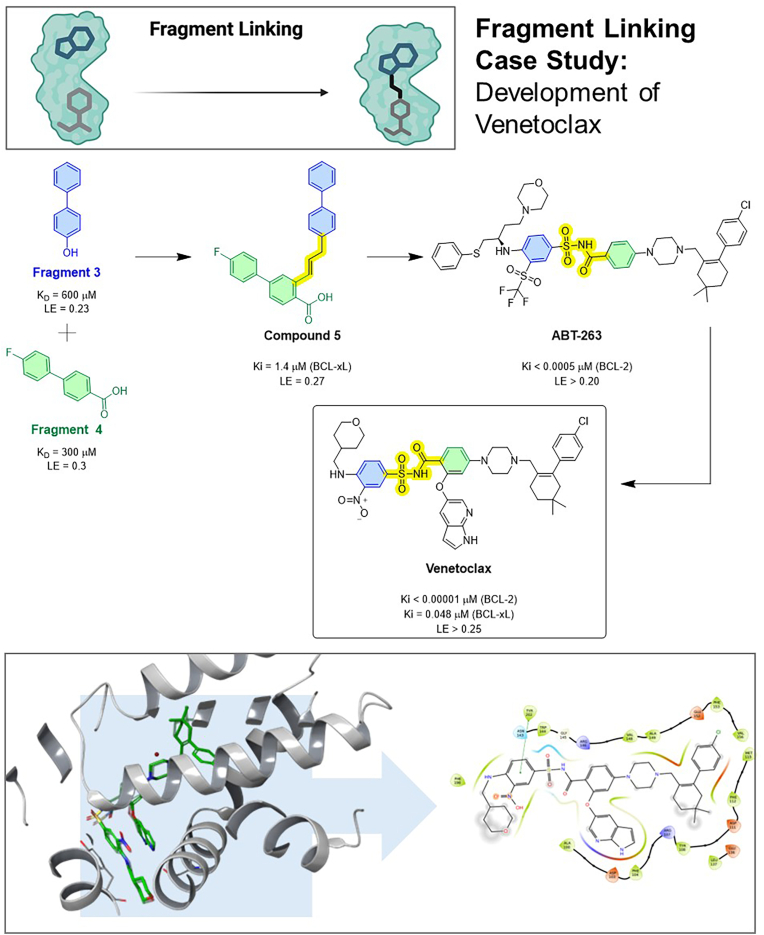
Fragment linking case study: The development of Venetoclax (ABT-199). This case study exemplifies the linking strategy for targeting the BCL-2 protein-protein interface. Two small fragments (Fragment **3** and Fragment **4**), identified by SAR by NMR, were found to bind to adjacent P2 and P4 pockets in the protein's BH3-binding groove. Guided by structural data, these low-affinity hits were connected with a linker (Compound **5**) and further optimised through intermediates like ABT-263. This culminated in Venetoclax, a highly potent and selective BCL-2 inhibitor. The crystal structure (PDB ID 6O0K) confirms the final molecule spans both original fragments binding sites, validating the strategy's success.

Achieved through fragment linking, where two fragments binding adjacent sites on BCL-2 were joined to create a highly potent and selective drug (Fig. 5). The anti-apoptotic protein BCL-2 was, for a long time, considered a difficult target (shallow protein–protein interface). Abbott scientists applied SAR by NMR in the early 2000s, identifying two small fragments (**3** and **4**) that bound distinct but adjacent pockets on BCL-XL (a homologue of BCL-2) (Petros et al., 2006; Souers et al., 2013). One fragment bound the P2 pocket, and another bound the P4 pocket of the protein's BH3-binding groove. Guided by NMR mapping and subsequently by crystal structures, they linked these fragments with an appropriate spacer (compound **5**). The linked compound ABT-263 showed low-nM affinity to BCL-XL/BCL-2. Further optimisation (to improve pharmacokinetics and selectivity for BCL-2) led to ABT-199 (Venetoclax). Venetoclax, approved in 2016 for chronic lymphocytic leukaemia (Deeks, 2016), became a breakthrough in targeting protein–protein interactions. Its success stems from the fragment linking strategy delivering a big jump in potency by combining weak binders. Subsequent crystal structures confirmed that Venetoclax occupies the BH3-binding groove, with its structure spanning the subsites initially targeted by each fragment (PDB ID 6O0K). This case also highlights an important lesson: fragment linking can produce larger molecules; Venetoclax is a relatively large (MW ∼868 Da) and hydrophobic compound, pushing the upper boundaries of oral drug-like properties. The development team succeeded by optimising metabolic stability and using linkers of minimal lengths. Venetoclax's development is a landmark in FBDD, demonstrating that even intracellular protein–protein interactions can be tackled by appropriately linking fragment hits.

### 6.3 KRAS-G12C inhibitor sotorasib (AMG 510)

**Fig. 6:**
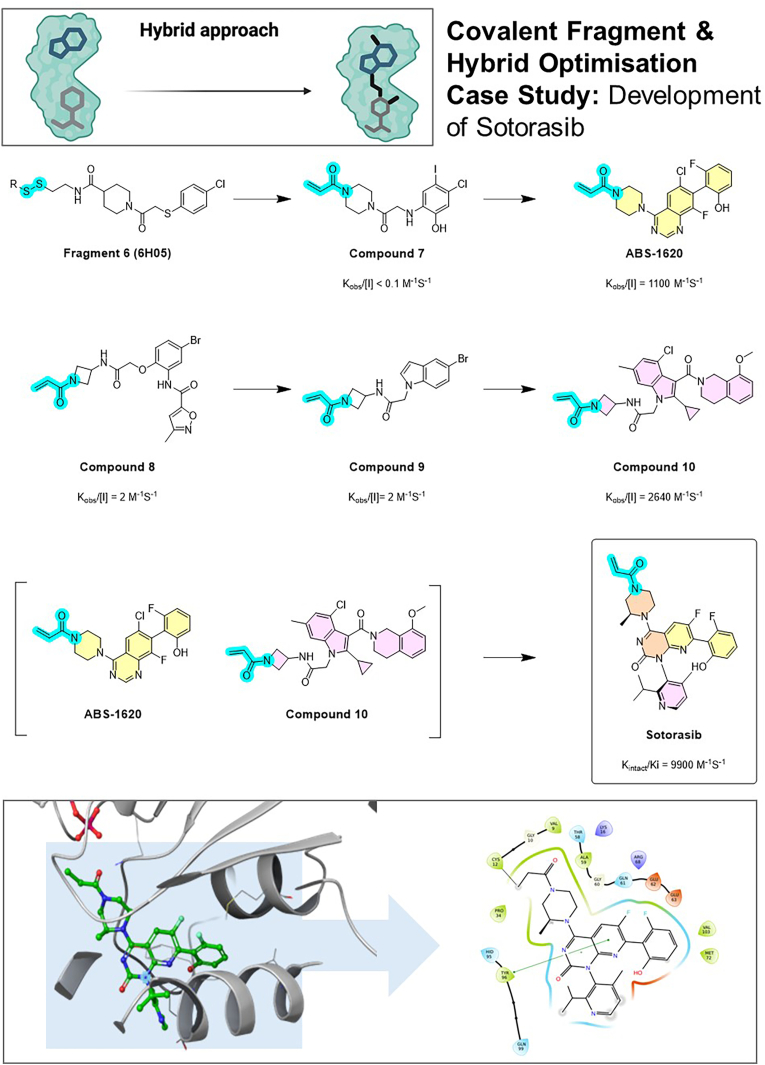
Covalent fragment and hybrid optimisation case study: The development of Sotorasib (AMG 510). This case illustrates a successful strategy against the historically “undruggable” KRAS G12C mutant. A cysteine-reactive fragment (Fragment **6**, 6H05) was identified that covalently binds to the mutant Cys12 residue in an inducible pocket. A hybrid optimisation approach, combining warhead optimisation (replacing a disulfide with a more suitable acrylamide; reactive electrophilic groups are highlighted in blue), fragment growing (yellow and pink rings; compounds ABS-1620 and **10**), and merging (orange rings; Sotorasib), was used to systematically elaborate this covalent anchor through a series of intermediates. This structure-guided process led to Sotorasib, a potent and selective inhibitor that became the first FDA-approved drug for this oncogenic target. The bottom panel shows the final compound bound in the KRAS G12C pocket (PDB ID 6OIM). (For interpretation of the references to colour in this figure legend, the reader is referred to the Web version of this article.)

Pioneered covalent fragment-based and hybrid optimisation (growing and merging) strategies, targeting a highly challenging oncogenic mutant (Fig. 6). KRAS G12C is a small GTPase mutant that was long deemed “undruggable”. FBDD played a pivotal role in finally cracking this target. In the mid-2010s, fragment screening (both covalent tethering and traditional) identified small cysteine-reactive fragments (**6** and **7**) that bind in an inducible pocket (the Switch-II groove) next to the mutant Cys12 (Ostrem et al., 2013; Mathieu et al., 2022). One such fragment was an acrylamide (**7**) that covalently bound Cys12 and occupied part of the pocket (Ostrem et al., 2013). Through structure-based design, fragments were grown (ABS-1620 and compound **10**) and merged to fill the adjacent pocket, while maintaining the covalent warhead. This led to potent inhibitors exemplified by AMG 510, known as Sotorasib (developed by FBDD) (Canon et al., 2019; Lanman et al., 2019) and MRTX849, known as Adagrasib (using a structure-based drug design approach) (Hallin et al., 2020), both approved in 2021–2022 for KRAS-mutated lung cancers (Rathod et al., 2023). These inhibitors are essentially larger molecules built from an initial fragment that was covalently tethered. The KRAS G12C project showcases how fragment methods (covalent fragment screening combined with traditional medicinal chemistry) opened up a novel binding site on a target previously considered inaccessible (Huang et al., 2021). It also underlines the value of integrating computational modelling: quantum mechanical modelling supported the design of optimal fragments and linkers to occupy the cryptic pocket. The success of Sotorasib has energised FBDD efforts against other challenging oncogenes.

## 7 Outlook

FBDD is rapidly evolving, with hybrid and orthogonal screening strategies (parallel NMR and crystallography, for example) now standard to maximise hit identification and confidence. Integration with HTS (“fragment-assisted drug discovery”) is increasingly common, using fragments to explore underrepresented chemical space and as building blocks to improve HTS hits (Varnes et al., 2016). Technological advances are pushing FBDD boundaries. Cryo-EM fragment screening and native mass spectrometry now enable exploration of previously inaccessible targets, such as membrane proteins and multi-protein complexes (Saur et al., 2020; Sternicki and Poulsen, 2024). Computational advances are accelerating optimisation cycles and predicting beneficial analogues (Zara et al., 2021; Yoo et al., 2025). For instance, FEP methods, have profoundly transformed fragment screening by enabling efficient, accurate *in silico* assessment of ligand binding affinities (Molani and Cho, 2024); distinguishing clearly between Relative Binding Free Energy (RBFE) for optimising analogues within known series (Cournia et al., 2017) and Absolute Binding Free Energy (ABFE) crucial for identifying and validating novel chemotypes (Alibay et al., 2022). This trend is being dramatically accelerated by new AI foundation models, such as Boltz2, which approach the accuracy of FEP for binding affinity prediction but are over 1000 times more computationally efficient (Passaro et al., 2025). This synergy between physics-based methods and AI-guided workflows promises to revolutionise the prioritisation and design of fragment analogues, with generative AI now emerging as a powerful tool for designing novel mergers and linkers (Filella-Merce et al., 2025).

Despite its successes, the FBDD field continues to address several key challenges. Reliably detecting very weak fragment binders with high confidence and low false-positive rates remains a primary technical hurdle (Cramer et al., 2017). The efficient optimisation of these low-affinity hits into potent drug candidates necessitates robust structural insights and innovative medicinal chemistry strategies, while constantly ensuring synthetic tractability. Furthermore, the design and curation of fragment libraries are an ongoing evolutionary process, aiming to maximise relevant chemical space coverage and include diverse, high-quality chemotypes (e.g., balanced sp^2^-rich/sp^3^-rich, 3D fragments) free of problematic features like PAINS (Bon et al., 2022). Additionally, covalent fragment screening (exemplified by KRAS G12C) has improved, enabling targeting of shallow or previously intractable pockets (Mathieu et al., 2022). Library design continues to expand beyond the “Rule of 3”, with interest in larger fragments (∼350 Da) and DEL to significantly increase the chemical space sampled (Luttens et al., 2025; Gironda-Martínez et al., 2021). Effectively tackling traditionally “undruggable” targets, particularly those with shallow or cryptic binding pockets, continues to demand new approaches.

The increasing adoption of an “orthogonal approach”, which combines experimental and computational techniques, is the practical manifestation and a hallmark of the FBDD advancement (Xu and Kang, 2025). This integration is not merely additive; it represents a powerful synergy that allows FBDD to effectively tackle targets previously considered “undruggable” or intractable. This convergence signifies a major leap in the capabilities of FBDD, enabling the field to move beyond traditional soluble protein targets to address complex biological systems (e.g., GPCRs, protein-protein interfaces), thereby significantly broadening the therapeutic landscape and opening new avenues for lead and drug discovery. Furthermore, new testing platforms, miniaturisation and automation serve as key drivers of throughput and accessibility (FitzGerald et al., 2024; Townley et al., 2023; Porta et al., 2025). This trend is critical for making FBDD more cost-effective, faster, and broadly accessible, particularly for academic laboratories or smaller biotechnology companies with limited resources. In the same direction, computational tools and AI/ML are significantly advancing the field, as illustrated by Lingo3DMol, a pocket-based 3D molecule generation method (Feng et al., 2024), their predictive accuracy and seamless integration into all stages of the FBDD/FBLD pipeline are areas of active development to further accelerate discovery cycles.

The impact of FBDD is evident in the rising number of clinical candidates (>50) across diverse therapeutic areas (such as oncology, antivirals, antibiotics, CNS diseases, and more) and has resulted in at least seven approved drugs: Vemurafenib (2011), Venetoclax (2016), Erdafitinib and Pexidartinib (both 2019), Asciminib and Sotorasib (both 2021), and Capivasertib (2023) (Khedkar et al., 2025). FBDD now underpins many integrated lead discovery strategies in pharma, complementing HTS, virtual screening, and structure-based design. Over the decade, FBDD will both benefit from and contribute to further advances in structural biology and computational chemistry, making the process faster, more predictive, and more powerful.

## 8 Conclusions

FBDD is a thriving and continuously advancing field that is delivering tangible results in drug discovery, pushing boundaries, and leveraging cutting-edge technologies to address previously intractable challenges. In addition, FBDD has redefined lead generation, empowering researchers to tackle challenging targets with efficient, high-quality starting points. Sensitive screening methods, robust optimisation strategies, and deep integration with structural biology and computational chemistry have led to multiple clinical candidates and approved drugs. FBDD enables the exploration of new chemical space, complements HTS, and is poised for even greater impact as methods and technologies advance. The trajectory of the field promises continual innovation and major therapeutic breakthroughs, truly validating the concept that “small” starting points can lead to significant advances in drug discovery.

## CRediT authorship contribution statement

Dana F. AlKharboush: Conceptualization, Visualization, Writing – review & editing. Frank Kozielski: Conceptualization, Funding acquisition, Supervision, Writing – review & editing. Geoffrey Wells: Conceptualization, Funding acquisition, Supervision, Writing – review & editing. Exequiel O. J. Porta: Conceptualization, Investigation, Methodology, Writing – original draft, Writing – review & editing.

## Funding sources

This work was supported by the 10.13039/100014013UK Research and Innovation – 10.13039/501100000265Medical Research Council (grant numbers MR/X013995/1).

## Declaration of competing interest

The authors declare that they have no known competing financial interests or personal relationships that could have appeared to influence the work reported in this paper.

## Data Availability

No data was used for the research described in the article.
